# Dispatching the wandering mind? Toward a laboratory method for cuing “spontaneous” off-task thought

**DOI:** 10.3389/fpsyg.2013.00570

**Published:** 2013-09-03

**Authors:** Jennifer C. McVay, Michael J. Kane

**Affiliations:** Department of Psychology, University of North Carolina at GreensboroGreensboro, NC, USA

**Keywords:** mind wandering, consciousness, goal priming, attention, current concerns

## Abstract

Cognitive psychologists and neuroscientists study most phenomena of attention by measuring subjects' overt responses to discrete environmental stimuli that can be manipulated to test competing theories. The mind wandering experience, however, cannot be locally instigated by cleverly engineered stimuli. Investigators must therefore rely on correlational and observational methods to understand subjects' flow of thought, which is only occasionally and indirectly monitored. In an effort toward changing this state of affairs, we present four experiments that develop a method for inducing mind wandering episodes—on demand—in response to task-embedded cues. In an initial laboratory session, subjects described their personal goals and concerns across several life domains (amid some filler questionnaires). In a second session, 48 h later, subjects completed a go/no-go task in which they responded to the perceptual features of words; unbeknownst to subjects, some stimulus words were presented in triplets to represent the personal concerns they had described in session 1. Thought probes appearing shortly after these personal-goal triplets indicated that, compared to control triplets, priming subjects' concerns increased mind wandering rate by about 3–4%. We argue that this small effect is, nonetheless, a promising development toward the pursuit of an experimentally informed, theory-driven science of mind wandering.

The scientific investigation of mind wandering is challenging, in part, because it is a subjective experience that must be assessed by self-report and validated against more objective, yet less direct, measures, such as task performance (e.g., Smallwood et al., 2008; He et al., 2011; McVay and Kane, 2012a), physiological or neural events (e.g., Smallwood et al., 2004; Christoff et al., 2009; Reichle et al., 2010; Uzzaman and Joordens, 2011), or cognitive-ability assessments (e.g., McVay and Kane, 2009; Mrazek et al., 2012; Unsworth and McMillan, 2013). An additional impediment to theoretical advance on mind wandering is that its study is inherently correlational: although laboratory studies have identified contextual manipulations that globally increase or decrease rates of task-unrelated thought (TUT) throughout a task (e.g., Antrobus et al., 1966; Teasdale et al., 1995; Forster and Lavie, 2009), investigators remain resigned to merely and occasionally observing the natural history of subjects' ongoing conscious experiences by periodically probing their thoughts. Until the field's methods allow for local intervention into the flow of thought, thereby initiating mind wandering episodes on demand and in response to theoretically motivated independent variables, mind wandering's causes and consequences will evade our understanding (McVay and Kane, 2010, 2012b; Smallwood, 2010, 2013). Here, then, we provide a progress report on the development of a (yet imperfect) method to locally initiate TUTs, which adapts priming techniques from the related research domains of involuntary autobiographical memory and motivated goal pursuit.

Before describing these priming techniques, we focus first on research suggesting that the flow of thought can be globally manipulated toward subjects' personal concerns, following from the everyday observation that surprising, stressful, or disturbing life experiences frequently intrude into consciousness. Indeed, one of the earliest laboratory investigations of mind wandering demonstrated empirically that news of geopolitical events having life consequences for subjects can influence their thought reports during ongoing tasks (Antrobus et al., 1966). College students sat in a waiting room before completing a vigilance task; for half the subjects, the mock radio broadcast playing in the background was interrupted by breaking news of an escalation of the Vietnam war and an impending acceleration of the military draft (control subjects heard no news report during the broadcast). During the subsequent vigilance task, thought probes periodically interrupted subjects to ask whether their preceding thoughts had been on-task or off. Unsurprisingly, subjects hearing the news about Vietnam reported more TUTs than did control subjects, and this difference remained constant throughout the 50 min task. Similar increases in global mind wandering rates occur after such emotionally evocative experiences as watching a disturbing, graphic film about Native Australian circumcision rites (Horowitz and Becker, 1971; Horowitz et al., 1971), watching a sad film about a gravely ill dog (Smallwood et al., 2009a) and, for young adults with stressful family relations, simulating a coercive confrontation with a parent (Klos and Singer, 1981).

Although such successful manipulations of TUT rates may rely partly on attendant increases in negative affect (see Smallwood et al., 2009a), the radio-broadcast and parent-confrontation conditions may also have worked because they oriented subjects toward their unresolved goals and plans for the future. According to Klinger's theory of thought content (which has influenced much of the recent empirical and theoretical work on mind wandering; e.g., Smallwood and Schooler, 2006; Mason et al., 2007; McVay and Kane, 2010; Smallwood, 2013), committing to a goal—whether as mundane as doing a load of laundry that evening or as lofty as completing an advanced educational degree—creates a latent state, or “current concern,” that sensitizes one to cues for opportunities to complete that goal (e.g., Klinger, 1971, 1999, 2009). Until that goal is fulfilled or abandoned, the current concern elicits a breadth of emotional, cognitive, and (if possible) behavioral responses upon encountering relevant cues, which help to mobilize the person toward goal fulfillment. Of primary relevance here, one such cognitive-emotional response to current-concern cues is the triggering of goal-related thoughts and images, which may represent mind wandering experiences if they happen to be unrelated to the ongoing task at hand.

A wealth of empirical evidence, from laboratory and daily-life studies, supports the contention of current concerns theory that thought flow is frequently oriented toward plans and unmet goals. For example, in laboratory studies that engage subjects in an ongoing cognitive task and ask them either to indicate via forced choice the temporal orientation of their TUTs (Smallwood et al., 2009b, 2011; McVay et al., 2013), or to give open-ended thought reports that are coded for temporal orientation by independent raters (Baird et al., 2011), the content of mind wandering episodes is most often oriented toward the future, rather than the present or past (see also Mason et al., 2008). In daily-life studies, as assessed via experience-sampling methods, subjects rate their mind wandering episodes as being oriented more toward “normal everyday things” (Kane et al., 2007) or “personal concerns or things I need to do” (McVay et al., 2009) than toward “worries” or “daydreams or fantasy”; moreover, current concerns that subjects explicitly endorse via questionnaires, prior to experience sampling, subsequently dominate their open-ended thought reports over the course of 24 h (Klinger et al., 1980).

More relevant to the method we developed for the present study, and perhaps more compelling than these observational studies of thought content, experimental manipulations of current concerns (à la Antrobus et al., 1966) significantly increase subjects' propensity to engage in concern-related thought, both while awake and while asleep. Nikles et al. (1998) asked subjects who spent several nights in a sleep laboratory to try to think all night about either one of their previously specified current concerns (on one night) or a yoked concern from another subject (on another night). Subjects provided dream reports upon being woken from each period of REM sleep, which were rated by blind reviewers for their associations to the subjects' concerns vs. yoked non-concerns. Experimenter-suggested current concerns were more frequent in dream reports than were non-concerns (and they increased with suggestion vs. two baseline nights), whereas experimenter-suggested non-concerns were actually slightly less frequent in dream reports than were subjects' current concerns.

Of course, night dreaming and daydreaming experiences are not the same (but for suggestive similarities, see Fox et al., 2013), and so it is most relevant here to note that mind wandering can also be nudged toward subjects' unfinished personal business. In a study that aimed to prime subjects' current life concerns, undergraduate subjects first wrote about either an important current goal and the steps they would take to complete it, or about a planned route to get from their current location to another campus building and what things they might see along that route (Stawarczyk et al., 2011). During a subsequent computer task, subjects characterized their immediately preceding thoughts on unpredictable probes and, if mind wandering, described the content of those TUTs. Concern-primed subjects did not mind wander more than did control subjects (nor did they explicitly think more often about their expressed goal), but they did have a higher proportion of TUTs that were independently rated as temporally oriented toward the future and as focused on future-oriented functions, such as planning and decision making. A stronger manipulation of subjects' thought content may rely on having them consider their goals *without* identifying means by which to fulfill them. Masicampo and Baumeister (2011) had subjects either write about two important, imminent, and non-routine tasks, or do the same while additionally specifying plans for completing each task. In a subsequent story-reading task, four thought probes assessed in-the-moment mind wandering and, following the task, subjects rated how distracted they had been by thoughts about their written-about goals. Subjects who had not yet committed to plans for their goals reported being more distracted by their goals while reading than were subjects who specified their plans; moreover, whereas 65% of the non-planners reported TUTs on at least one thought probe, only 33% of the planners did.

Although limited in number, the studies reviewed above are promising, insofar as they indicate that mind wandering rate and thought content may be globally influenced by experimental manipulations. That is, task-wide TUT rates can be driven up or down, on average, when subjects are previously cued vs. not cued to consider (without planning) their unfulfilled personal goals and concerns. Such broad manipulations, however, are of limited value for testing competing theories of mind wandering, as they leave ambiguous the immediate causes of particular TUTs for particular subjects at particular times (Smallwood, 2013); indeed, mind wandering studies usually have no way of discriminating the number of times a subject mind wanders (i.e., TUT frequency) from the length of time a subject typically spends mind wandering (i.e., TUT duration), as both yield similarly high probed TUT rates. Smallwood (2013) nicely illustrates the plight of theory in the mind wandering domain by noting the role of the imperative stimulus in most other studies of cognition: investigations of “mainstream” attentional phenomena such as repetition blindness, the attentional blink, visual search, selective filtering, response competition, task-set switching, etc., all assess subjects' responses to discrete stimuli presented at carefully timed intervals. Theorists can thus make relatively strong claims about the proximal causes and consequences of the behaviors they observe. So, although it may be somewhat helpful to know that subjects will mind wander more during a 20 min vigilance task if they have previously written about their personal concerns, theorists are still left uncertain about how or why subjects' control over thought waxes and wanes throughout the task. They also cannot know how or why a particular subject experiences a TUT at one moment rather than another (or experiences a TUT about one topic or another, or experiences many brief TUTs vs. few long TUTs).

It is, therefore, scientifically imperative that investigators develop some kind of imperative stimulus for mind wandering. Our present attempt to do so—that is, to create stimuli that might initiate TUTs on demand—was built largely upon prior work by Klinger and others on priming people's current concerns within the ongoing flow of thought [Hoelscher et al., 1981; Nikula et al., 1993; see also Klinger, unpublished, described in Klinger (1978)]. The common thread through this research is that subjects first describe several of their current goals and concerns via questionnaire or interview assessment, and then these concerns are translated by the researcher into short phrases (e.g., “*DOCTOR—LIFELONG—AMBITION”; “ROOMATE—THREATENS—EXISTENCE*”; Nikula et al., 1993) which are then inserted as concern-related cues within a subsequent task or activity. These studies show that: (1) current-concern cues inserted into a Stroop task interfere with color naming more than do non-concern-related words (Riemann and McNally, 1995); (2) current-concern cues played aloud as subjects enter REM sleep influence immediate dream-report content more than do non-concern-related words (Hoelscher et al., 1981); (3) current-concern cues played aloud as subjects sit listening to them increase skin conductance compared to non-concern cues and baseline recordings (Nikula et al., 1993); (4) and, of most relevance, current-concern cues played over headphones while subjects engage in dichotic listening of literary passages increase probed thought reports of concern-related thoughts vs. non-concern cues, and increase the time spent listening to the passage/channel in which the concern cues were just presented [in Klinger (1978)]. It appears, then, that concern-related words and phrases have the power to cue conscious thoughts to those concerns, at least when subjects are not otherwise engaged in a very demanding task or activity.

Our optimistic speculation, that priming subjects' current concerns with short phrases might serve as an effective trigger for TUTs, is supported further by recent work on spontaneous autobiographical memory (the experience of suddenly remembering a past episode from one's life without having deliberately retrieved it). Daily-diary studies of spontaneous autobiographical memories indicate that subjects are typically aware of an external cue that triggered the recollective experience (e.g., Bernsten and Hall, 2004; Kvavilashvili and Mandler, 2004; Ball and Little, 2006), and laboratory studies have found that spontaneous autobiographical memories can be cued, in-the-moment, by visual distractors. Schlagman and Kvavilashvili (2008; see also Kvavilashvili and Schlagman, 2011) had subjects complete a perceptual vigilance task that also presented irrelevant, to-be-ignored phrases on-screen, each representing a potential setting that subjects may associate with a previous life event (e.g., *relaxing on a beach; missed opportunity; crossing the road*). Any time subjects experienced a spontaneous autobiographical memory, they hit a key (to record the precise time) and described and rated the memory along various dimensions. The key result was that, across multiple experiments, about 85–90% of reported memories were identified by subjects as being triggered by something in the environment; 85–90% of these instances were identified as having been cued by an onscreen phrase. On average, subjects pressed the key 5 s after the triggering cue phrase appeared, and this time was considerably less than in a deliberate-retrieval condition, where these same subjects attempted to recall an autobiographical memory to each phrase cue (with an average of about 10 s).

In summary, then, conscious thoughts and experiences appear to be influenced by environmental cues that subjects associate with their unfulfilled goals and ongoing personal concerns (Klinger, 1971, 1999, 2009). We therefore sought to harness this cue-sensitive dimension of thought flow as a means to experimentally cause TUT experiences on demand, in response to discrete stimulus events (i.e., local cues to subjects' personal concerns). Our hope was that such a method may eventually allow investigators of mind wandering to make the kind of causal claims permitted in other domains of attention and consciousness research, where the timing and content of subjects' responses and experiences can be empirically grounded to identifiable and manipulable environmental events.

We adapted the local cuing methods described above while also attempting to reduce demand characteristics: many of those concern-priming and spontaneous-memory-priming studies presented cues that were obvious to the subjects (i.e., focally attended), and so the connection between the cues and the current-concern assessments they had previously completed may have also been salient to them. If subjects make the explicit connection between their prior goal reports and the concern cues embedded in the task, they may be biased to report thoughts that are consistent with the investigators' hypotheses. To minimize such demand, we assessed subjects' current concerns in a separate session from the concern-cuing task, we embedded this current-concern assessment amid a number of other questionnaires to mask its significance, and we presented current-concern cues in a task context that did not require subjects to process the meanings of those cues (i.e., we attempted to prime subjects' concerns surreptitiously and relatively implicitly during the primary task).

Our primary hypothesis, tested in the four experiments reported here, was that by indirectly cuing individual subjects' personal goals and concerns with three-word-phrases within the context of an ongoing vigilance task, we would increase TUT reports following those cues (compared to cuing by non-concerns). Our secondary hypothesis was that, by cuing TUTs with concern-related cues, we would also increase performance errors on trials following those cues.

## General method

Our Methods sections will report how we determined our sample size, all data exclusions, all manipulations, and all measures in the study (Simmons et al., 2012).

### Subjects

Undergraduates at the University of North Carolina at Greensboro (UNCG) participated in each experiment, tested in groups of 1–6, in partial fulfillment of a psychology course requirement. Our goal for each experiment was to test as many participants as possible within a single academic semester (aiming for *N* = 60), under the pragmatic scheduling restriction that the first author had to serve as the experimenter for the second data-collection session for all subjects (see below). Thus, our stopping rule for data collection was the end of the semester; we did not determine sample size by periodically exploring the data. Sixty-three students participated in Experiment 1, 64 in Experiment 2, 57 in Experiment 3 and 67 in Experiment 4. The numbering of experiments reflects their chronological sequence (i.e., Experiment 1 was conducted first, Experiment 4 last).

#### Procedure

In all four experiments, we attempted to covertly cue mind wandering episodes during a long-duration Sustained Attention to Response Task (SART; Robertson et al., 1997; McVay and Kane, 2009) using a two-session procedure. Subjects attended two sessions within 1 week (scheduled 2 days apart; e.g., Monday-Wednesday), each conducted by a different experimenter in order to mask the connection between sessions. All second sessions in Experiments 1–4 were conducted by the first author. During Session 1, subjects completed a battery of surveys; the primary purpose was to collect information about subjects' personal goals and concerns. Session 2 required subjects to complete a computerized SART with embedded thought probes to assess mind wandering. Between sessions, the Session 2 experimenter generated three-word cues from the subject's expressed personal concerns and inserted them as consecutive SART stimuli. We did not tell subjects that the sessions were meaningfully connected until a debriefing at the end of Session 2.

### Stimuli and materials

#### Session 1 surveys

Subjects completed four questionnaires, in this order: (1) Cognitive Failures Questionnaire—Memory and Attention Lapses (CFQ-MAL; McVay and Kane, 2009; available from http://www.uncg.edu/~mjkane/memlab.html); (2) Personal Concerns Inventory (PCI; adapted from Cox and Klinger, 2004; Klinger and Cox, 2004); (3) an adult-self-report version of the AD/HD Rating Scale (DuPaul et al., 1998), and; (4) Action Orientation Scale (AOS; Kuhl, 1985). All were completed on paper in Experiments 1–3, whereas the CFQ-MAL and AOS were completed via computer in Experiment 4. The main purpose of including the CFQ-MAL, AD/HD Rating Scale, and AOS was to deflect subjects' focus from the PCI, particularly during Session 2, as we hoped to cue their personal concerns *indirectly* in that session in order to avoid demand characteristics. However, because some of the content of these surveys was conceptually related to mind wandering experiences and absentminded errors, we will report their data in a “*post-hoc* analyses” section.

The primary measure—from our perspective—the PCI, presented nine categorical prompts (e.g., interpersonal relationships; finances and employment) and asked subjects to provide a written description of a related goal or concern for each, along with estimating when they expected to resolve the goal or concern (e.g., “*I am worried about seeing my sister at Thanksgiving because we haven't spoken in months [2 months]*.”). After completing each prompt, the experimenter instructed subjects to rate each response for importance on a 1–10 scale, with 1 representing “*not at all important to me*” and 10 representing “*most important goal or concern in my life right now*.” The Session 2 experimenter sought the most important and imminent concerns for the mind wandering cues embedded in the SART.

#### SART

We adapted the SART from the “perceptual” version used by McVay and Kane (2009) because it encouraged subjects to respond to stimulus words based on their orthographic features rather than their meanings; this method allowed us to cue subjects' personal concerns more implicitly via embedded goal-related words. Subjects pressed the space bar as quickly as possible for lowercase words (non-target “go” trials) and withheld responding to infrequent uppercase words (target “no-go” trials). Each stimulus word appeared for 300 ms followed by a 900 ms mask. Thought probes appeared throughout the task, asking subjects to classify their immediately preceding thoughts as follows: (1) task; (2) task performance; (3) everyday stuff; (4) current state of being; (5) personal concerns; (6) daydreams; (7) other. The experimenter explained and clarified each of these thought categories during SART instructions (see McVay and Kane, 2009). We coded responses 3–7 as TUTs for all analyses.

The Session 2 experimenter individually prepared each subject's SART based on that subject's PCI responses. Specifically, the experimenter chose two of the subject's personal concerns based on importance ratings, selecting the two highest-rated concerns as long as they were somewhat specific to the subject (e.g., not “graduate college,” as all the subjects were college students). If a subject claimed more than two highly rated goals, the experimenter chose the most imminent. Finally, the experimenter used apparent specificity or uniqueness of the goal to select among equally important and imminent concerns. Once the experimenter settled on the particular concerns to use for each subject, she created a three-word phrase to capture its meaning while also attempting to use different vocabulary from the subject, when possible. For example, the concern, “*I want to grow a beard*,” was translated into *INCREASE—FACIAL—HAIR*, avoiding the subject's words, “grow” and “beard” while retaining the meaning. Our goal was to minimize explicit recognition of the PCI-SART connection while still effectively cuing subjects' personal goals and concerns.

The experimenter-generated cues to each subject's personal goals and concerns appeared as three consecutive non-target SART stimuli (appearing a specified number of trials preceding each no-go target, depending on the experiment). We also created a single set of control word triplets to mirror the personal-concern cue words for all subjects (with the exception of Experiment 1; see below). The details of the placement of both the cues and the thought probes within the SART varied between experiments, as specified below.

Each SART was divided into blocks of 135 trials, with each block comprising a set of 45 trials that repeated three times in succession, and presenting 15 target no-go trials (11% of all trials). The division of trials into sets and blocks was seamless to the subjects. The personal concern (PG) word triplets and the control (“other goal”; OG) word triplets each appeared once per set of 45 trials (the “other goal” triplets for Experiments 2–4 were taken from actual subjects' goals from Experiment 1: *INCREASE—FACIAL—HAIR; COMPENSATE—PIANO—PLAYER; WART—REMOVAL—TREATMENT; WASH—TWO—PETS*). For each subject, the two personal-concern word triplets (PG1 and PG2) and two corresponding control triplets (OG1 and OG2) alternated between blocks (e.g., PG1 and OG1 in Block 1, PG2 and OG2 in Block2). The Appendix specifies the exact placement of all concern cues, targets, and thought probes for each experiment.

#### Post-SART questionnaire

A final questionnaire asked subjects about the task stimuli, the connection between the sessions, and their mind wandering experiences. The open-ended questions asked subjects: (1) what the connection between the two sessions might have been; (2) whether they recalled any particular words that “stood out” to them from the SART; and (3) about the contents of their thoughts if they selected “other” on a thought probe. Subjects then rated their concentration and effort in the SART on 7-point scales.

Subjects were then asked specifically about the concerns that we used to generate the cues in their SART task. We asked them to report (yes/no) whether they recalled ever thinking about the concern (presented in the subject's own words) during the SART and, if yes, how frequently they did so and which probe option they selected at the time. Finally, subjects were asked to rate the experimenter-generated cue words used in their task (both their personal-concern cue words and the control cue words) for their relatedness to the subjects' own personal concerns on a 7-point scale with 1 being “*not at all related*” to 7 being “*perfect match*.”

## Experiment-specific SART methods

### Experiment 1

The SART presented 810 trials, divided into 6 blocks of 135 trials (sets of 45 trials repeated 3 times in succession) with 15 target trials per block. The word triplets each appeared three times per block at a distance of 1, 3, or 5 non-targets before a target trial that was followed immediately by a thought probe.

In only Experiment 1, we used two sets of control word triplets. “Yoked goal” triplets were generated, for each subject, from some of the PG cue words from *other* subjects tested in their session (e.g., if one subject reported, “*I need to talk to my sister about travel for Thanksgiving*” on the PCI, then “*SIBLING—HOLIDAY—PLANS*” might appear as a PG cue for that subject and as a yoked-goal cue for another subject). The Session 2 experimenter chose the yoked-goal cues by shuffling PCIs and pairing subjects in a pseudo-random way; although the experimenter checked that the two subjects did not report overlapping goals on the PCI, this could not completely account for subjects' unreported concerns, as the PCI is not exhaustive. We designed OG word triplets with similar structure and repetitions to PG goal triplets, but these cues did not represent any subjects' concerns (i.e., *CLOSE—WOODEN—DOOR*; *SWEEP—UNDER—RUG*).

Subjects' responses to the post-SART questionnaire indicated that the yoked-goal cues were often closely related to the subjects' own goals (which is, perhaps, unsurprising given the similarities among the undergraduate subjects' concerns, in general). Therefore, we used the OG triplets for Experiment 1 analyses. As previously indicated in the General Methods, Experiments 2–4 used one set of four control OG triplets for all subjects.

### Experiment 2

The SART presented 1080 trials, divided into 8 blocks of 135 trials (sets of 45 trials repeated 3 times in succession) with 15 target trials per block. All the PG and OG word triplets appeared at a distance of only 1 non-target trial before a target trial that was followed immediately by a thought probe. In this experiment only, additional thought probes were attached to non-target trials, but for the sake of continuity with the other experiments, we did not analyze them here.

### Experiment 3

As in Experiment 2, the SART presented 1080 trials, divided into 8 blocks of 135 trials (sets of 45 trials repeated 3 times in succession) with 15 target trials per block; as well, each PG and OG word triplet appeared 1 non-target trial before a target trial that was immediately followed by a thought probe. (No probes followed non-target trials).

### Experiment 4

As in Experiment 1, the SART presented 810 trials, divided into 6 blocks of 135 trials each (sets of 45 trials repeated three times in succession) with 15 target trials per block. The PG and OG word triplets appeared three times per block at a distance of 1, 3, or 5 non-targets before a target trial that was followed immediately by a thought probe.

## Results

**Figure 1:**
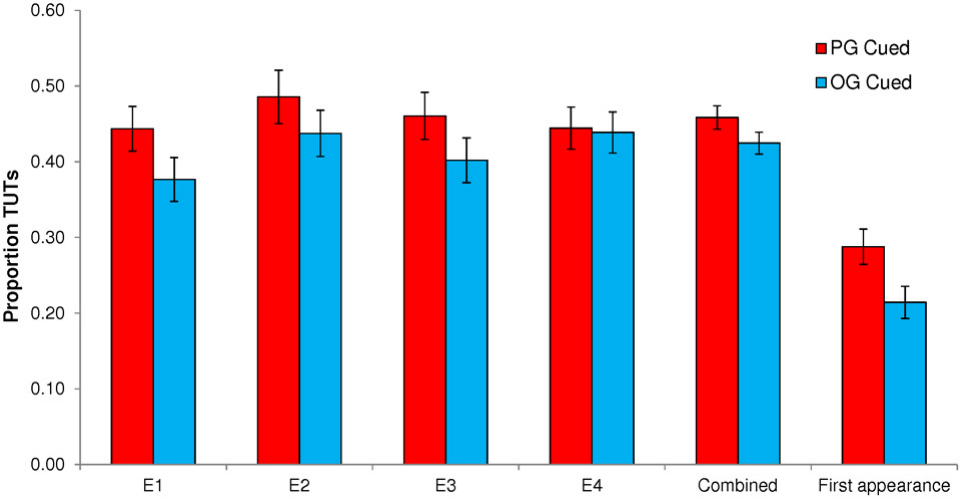
**Proportion of task-unrelated thoughts (TUTs) in the Sustained Attention to Response Task following personal-goal (PG) cues and other-goal (OG) cues, for Experiments 1–4 (E1–E4) individually, for the full dataset combined across experiments, and for the first appearance only of each PG and OG cue combined across experiments**. Error bars represent standard errors.

**Figure 2:**
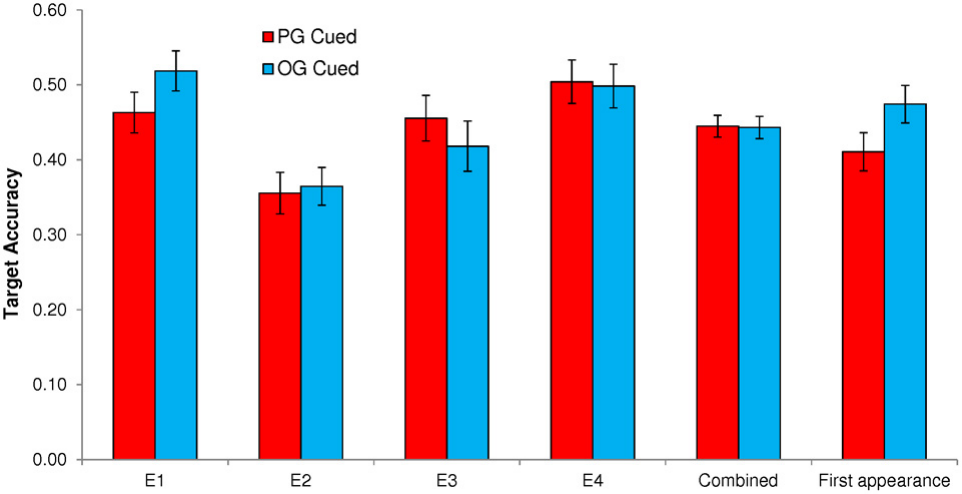
**Target accuracy rates in the Sustained Attention to Response Task following personal-goal (PG) cues and other-goal (OG) cues, for Experiments 1–4 (E1–E4) individually, for the full dataset combined across experiments, and for the first appearance only of each PG and OG cue combined across experiments**. Error bars represent standard errors.

Figure 1 presents mean probe-caught TUT rates for PG- vs. OG-cued trials by Experiment, and Figure 2 presents mean accuracy rates on no-go SART targets by Experiment.

### Mind wandering

To preview our primary findings—and as is evident in Figure 1—thought probes that followed PG cue triplets yielded modestly higher TUT rates than did those that followed OG cue triplets in three of our four experiments (and in a combined-experiment analysis).

#### Experiment 1

**Figure 3:**
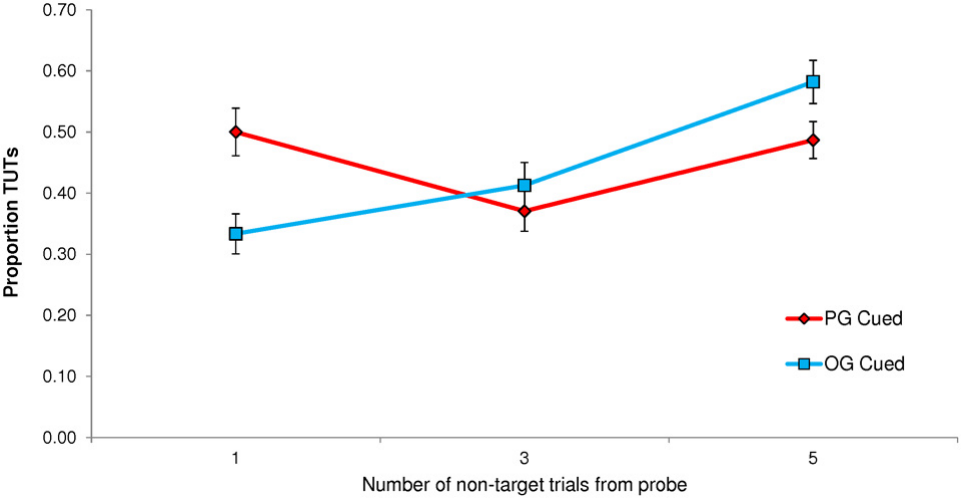
**Experiment 1 proportion of task-unrelated thoughts (TUTs) in the Sustained Attention to Response Task following personal-goal (PG) cues and other-goal (OG) cues, presented either 1, 3, or 5 non-target trials preceding the target and thought probe**.

A paired *t*-test indicated that TUT rates were significantly higher following PG cues (*M* = 0.444; *SD* = 0.234) than OG cues (*M* = 0.377; *SD* = 0.229), *t*_(62)_ = 3.94, *p* < 0.001. This TUT-rate increase of *M* = 0.067, 95% CI [0.033, 0.101], for PG cues reflected a moderate effect size (*d* = 0.497). In order to determine whether cue-to-probe distance affected personal goal cuing (PG and OG cues appeared 1, 3, or 5 non-target trials prior to the target event and probe), we conducted a Cue × Distance repeated measures ANOVA. It did: the interaction was significant, *F*_(2, 124)_ = 13.36, *p* < 0.001, MSE = 0.604, η^2^_*p*_ = 0.177. As depicted in Figure 3, PG cues only prompted more TUTs than did OG cues when they appeared 1 non-target trial away from the probe. (This finding led us to use only a cue-to-non-target distance of 1 in Experiments 2 and 3).

#### Experiment 2

As in Experiment 1, PG cues elicited significantly higher TUT rates (*M* = 0.486, *SD* = 0.282) than did OG cues (*M* = 0.438, *SD* = 0.244), *t*_(63)_ = 2.03, *p* = 0.046. Here, however, the TUT-rate increase of *M* = 0.048, 95% CI [0.001, 0.096] following PG cues reflected a smaller effect size (*d* = 0.259).

#### Experiment 3

As in Experiments 1 and 2, PG cues produced significantly elevated TUT rates (*M* = 0.461, *SD* = 0.234) compared to OG cues (*M* = 0.402, *SD* = 0.223), *t*_(56)_ = 3.43, *p* = 0.001. The TUT-rate difference between PG and OG conditions here, *M* = 0.059, 95% CI [0.025, 0.094], reflected a moderate effect size (*d* = 0.457) that more closely resembled that from Experiment 1.

#### Experiment 4

**Figure 4:**
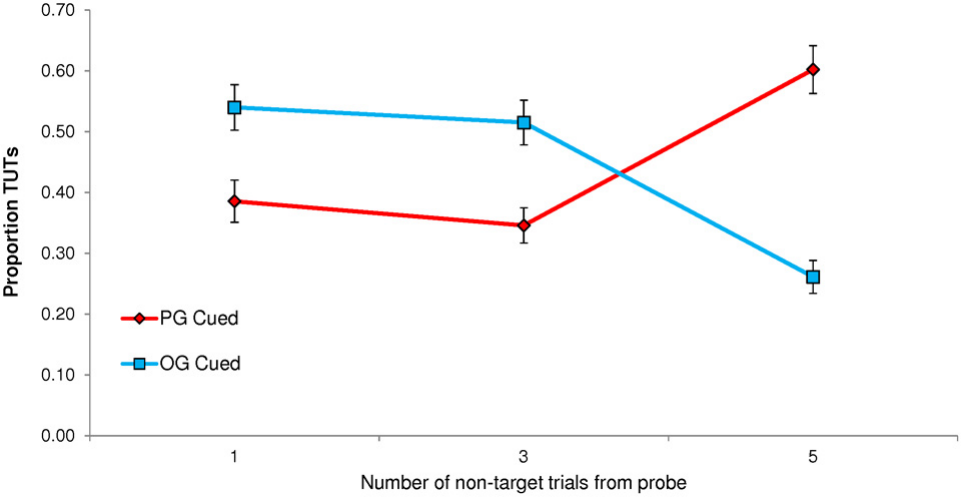
**Experiment 4 proportion of task-unrelated thoughts (TUTs) in the Sustained Attention to Response Task following personal-goal (PG) cues and other-goal (OG) cues, presented either 1, 3, or 5 non-target trials preceding the target and thought probe**.

Unlike the previous experiments, Experiment 4 did not yield a significant effect on TUT reports of PG cues (*M* = 0.444, *SD* = 0.228) vs. OG cues (*M* = 0.439, *SD* = 0.222), *t*_(66)_ = 0.38, *p* = 0.778; the corresponding effect size of this difference, *M* = 0.005, 95% CI [−0.026, 0.036], was trivial (*d* = 0.046). As we did for Experiment 1, where cues also appeared at different distances from the target and thought probe, we conducted a Cue × Distance repeated measures ANOVA to determine whether the cue-to-probe distance affected PG vs. OG cuing. It did, *F*_(2, 132)_ = 51.14, *p* < 0.001, MSE = 2.821, η^2^_*p*_ = 0.437, but the results did not mirror those from Experiment 1. As Figure 4 illustrates, PG cues elicited more mind wandering than did OG cues only when they appeared 5 non-targets away from the probe; moreover, OG cues appeared to elicit more TUTs than did PG cues at distances of 1 and 3.

#### Combined-experiment and meta-analyses

**Figure 5:**
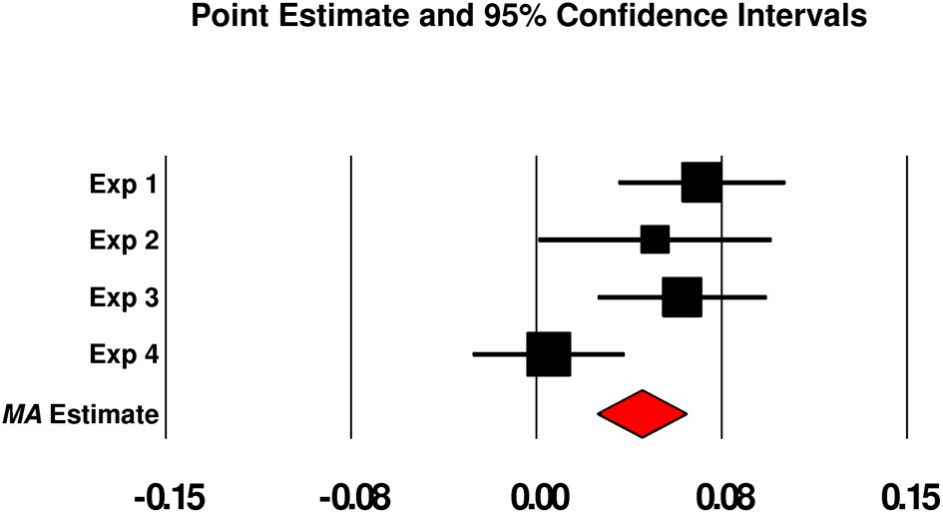
**Forest plot from a fixed-effect meta-analysis of the point estimate for the differences in mind wandering rates between personal-goal cued trials and other-goal cued trials across Experiments 1–4**. Boxes represent the weighted point estimates for each experiment, error bars represent 95% confidence intervals, and the red diamond represents the meta-analytic point estimate across all experiments (with diamond width reflecting the 95% confidence interval). Note: Exp 1, Experiment 1; Exp 2, Experiment 2; Exp 3, Experiment 3; Exp 4, Experiment 4; MA estimate, meta-analytic point estimate.

A preponderance—but not a totality—of evidence across experiments indicated that cuing subjects with their personal concerns during the SART led to a subtle increase in mind wandering relative to control (other-goal) cuing. We therefore, sought to increase our power to detect a significant effect of PG vs. OG cuing on TUT rates (and to protect against a false positive inference; Schimmack, 2012), as well as to generate a more accurate point estimate for the size of any such effect. We did so, first, by combining the data across experiments (*N* = 251), a reasonable approach given how similar the SART and thought-probe methods were across experiments. As is suggested by Figure 1, a paired *t*-test indicated that, in the combined dataset, PG cues significantly increased TUT rates on subsequent probes compared to OG cues (*M*s = 0.458 vs. 0.425, *SD*s = 0.245, and 0.231, respectively), *t*_(250)_ = 3.63, *p* < 0.001. This mean difference of 0.033 [95% CI = 0.015, 0.052] between PG- and OG-cued TUT rates reflected a small effect size (*d* = 0.230). Second, we conducted a fixed-effect model meta-analysis of Experiments 1–4, the results of which are presented in the Figure 5 forest plot. The meta-analytic point estimate for the TUT-rate difference between PG- and OG-cued trials was.042 [95% CI = 0.024, 0.060]^1^.

### No-go target accuracy

Our secondary question was whether any PG-cuing effect might be strong enough to not only induce mind wandering, but also to derail subjects' train of thought sufficiently to affect their SART performance accuracy. To preview our findings, the effects of PG vs. OG cues on SART accuracy were highly inconsistent across the four experiments, as can be seen in Figure 2. Only one experiment produced the expected pattern of PG cuing reducing the successful withholding of responses to no-go targets, and a combined-experiment analysis suggested no cuing effect, overall.

#### Experiment 1

**Figure 6:**
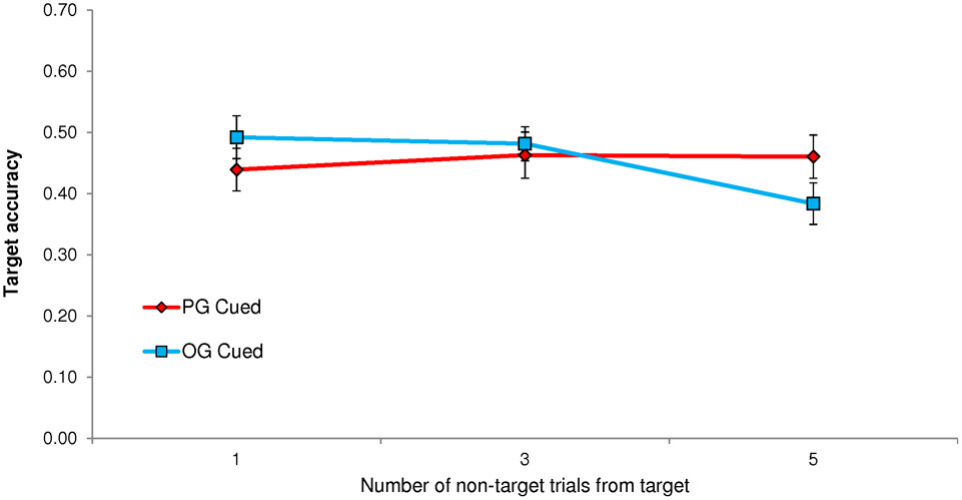
**Experiment 1 accuracy rates in the Sustained Attention to Response Task following personal-goal (PG) cues and other-goal (OG) cues, presented either 1, 3, or 5 non-target trials preceding the target and thought probe**.

PG cues had a statistically significant effect on target accuracy, *t*_(62)_ = −2.87, *p* = 0.006, whereby subjects were significantly less accurate on target trials following PG cues (*M* = 0.463, *SD* = 0.216) than OG cues (*M* = 0.519, *SD* = 0.212). This accuracy difference, *M* = −0.056, 95% CI [−0.094, −0.017], reflected a small-to-moderate effect size (*d* = −0.362). A Cue × Distance repeated measures ANOVA indicated a significant interaction that paralleled that for TUT rate, *F*_(2, 124)_ = 3.48, *p* = 0.034, MSE = 0.142, η^2^_*p*_ = 0.053, indicating poorer target accuracy following PG vs. OG cues only when those cues appeared 1 non-target trial before the target (see Figure 6).

#### Experiment 2

Target accuracy was not significantly impaired following PG cues (*M* = 0.356, *SD* = 0.221) vs. OG cues (*M* = 0.365, *SD* = 0.202), *t*_(63)_ −0.38, *p* = 0.706. Although the numerical difference between these conditions was in the predicted direction (as in Experiment 1), *M* = −0.009, 95% CI [−0.057, 0.039], the effect was trivial (*d* = −0.047).

#### Experiment 3

In contrast to both Experiments 1 and 2, no-go accuracy following PG cues (*M* = 0.455, *SD* = 0.229) was significantly *higher* than following OG cues (*M* = 0.418, *S*D = 0.253), *t*_(57)_ = 2.30, *p* = 0.025; this accuracy improvement with PG cuing, *M* = 0.037, 95% CI [.004,.069], reflected a small-to-moderate effect size (*d* = 0.312) in the non-predicted direction.

#### Experiment 4

**Figure 7:**
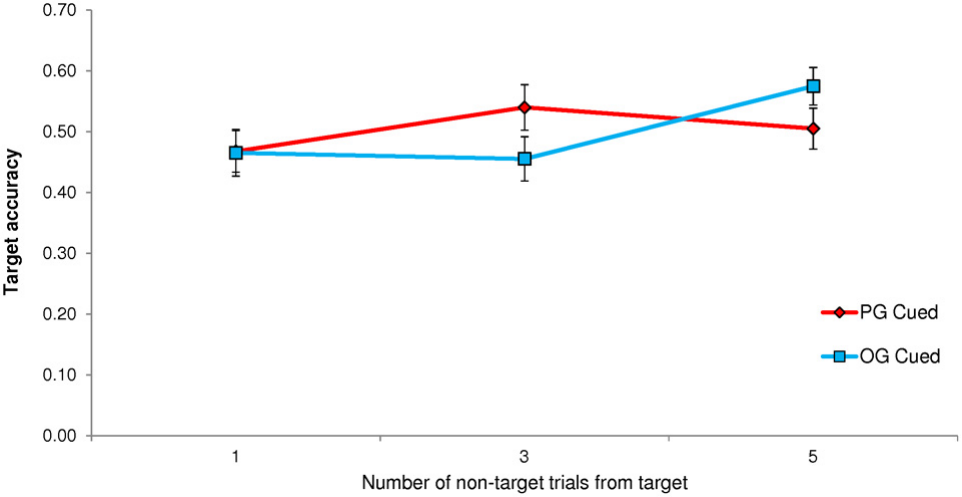
**Experiment 4 accuracy rates in the Sustained Attention to Response Task following personal-goal (PG) cues and other-goal (OG) cues, presented either 1, 3, or 5 non-target trials preceding the target and thought probe**.

As in Experiment 2, PG-cued trials (*M* = 0.504, *SD* = 0.238) did not differ significantly in accuracy from OG-cued trials (*M* = 0.498, *SD* = 0.239), *t*_(66)_ = 0.31, *p* = 0.755; this accuracy difference in the non-predicted direction, *M* = 0.006, 95% CI [−0.031, 0.043], reflected a very small effect (*d* = 0.038). A Cue × Distance repeated-measures ANOVA on target accuracy indicated a significant interaction that resembled that for TUT rates, *F*_(2, 132)_ = 4.60, *p* = 0.011, MSE = 0.199, η^2^_*p*_ = 0.065. That is, as illustrated in Figure 7, PG target accuracy was somewhat lower than OG target accuracy only when the cues appeared 5 non-target trials before the target (distance 3 appears to have yielded the opposite pattern).

#### Combined-experiment and meta-analyses

As we did for our TUT-rate analyses, here we first combined the SART accuracy data across all four experiments (*N* = 251; see Figure 2). As expected from the individual experiments' analyses, the PG-cued accuracy rate for no-go targets (*M* = 0.445, *SD* = 0.232) did not differ significantly from the OG-cued rate (*M* = 0.443, *SD* = 0.234), *t*_(250)_ = 0.17, *p* = 0.866, and this PG-OG accuracy difference, *M* = 0.002, 95% CI [−0.018, 0.022], represented a miniscule effect in the non-predicted direction (*d* = 0.011). We next conducted a fixed-effect model meta-analysis of Experiments 1–4: consistent with the combined-experiment analysis above, the meta-analytic point estimate for the accuracy-rate difference between PG- and OG-cued target trials was a negligible −0.002 [95% CI = −0.020, 0.018].

### Post-SART questionnaire responses

In general, relatively few subjects seemed aware of the true connection between the initial questionnaire session (i.e., the PCI assessment of current concerns) and the SART session, and so our main findings are not likely to have been contaminated by demand characteristics. Across Experiments 1–4, respectively, only 6, 8, 4, and 6% of subjects made any reference to the PCI or personal goals and concerns when asked about how the two sessions might have been related. Indeed, in each experiment, most subjects guessed that both sessions were aimed at assessing AD/HD in some way (recall that one of the Session 1 measures was an AD/HD questionnaire).

A minority of subjects recalled at least one of the PG-cue words when asked to recall any specific words they had seen in the SART (33, 41, 23, and 29%, respectively, in Experiments 1–4). But, when provided with their original descriptions of their two PG-cued concerns, a majority of subjects reported having thought about those concerns at some point during the SART (63, 67, 60, and 54%, respectively, in Experiments 1–4). On a 7-point scale of relatedness to the subject's own personal concerns, the PG word-triplet cues were given mean ratings of 6.27, 6.00, 6.18, and 5.99 across Experiments 1–4, respectively, and the OG cues were correspondingly rated with means of 2.83, 2.03, 2.12, and 2.19. In all four experiments, mean ratings (on a 1–7 scale) for subjects' overall “concentration” and “effort” in the SART were fairly tightly distributed, a bit above the mid-point, ranging from a low of 4.60 (Experiment 1 “concentration”) to a high of 4.92 (Experiment 2, “effort”).

### Exploratory *post-hoc* analyses

#### Novelty of PG cue triplets

Our hypothesis-driven, planned analyses that were reported above found that PG cues had a small-but-significant effect on TUT rates, with more mind wandering reports following PG cues than OG cues, but also that PG cues had no consistent effect relative to OG cues on downstream accuracy to no-go targets. Here we assessed whether our priming method may have been limited by repeating PG and OG cue words throughout the task. Perhaps PG word cues can lose some of their salience with repetition, or perhaps cuing two different personal goals in alternation throughout a task disrupts the power of each to affect thought. To examine this potential limitation, we combined the data across experiments and isolated the very first exposure to each PG and OG cue triplet to assess their effects on TUTs and accuracy. That is, we created a new dataset using only the first appearance of each of the two PG and two OG cue triplets, and the corresponding TUT and accuracy data from the nearest subsequent target trial and probe. Each subject, therefore, contributed two data points for PG cues and two for OG cues (the relevant trials are bolded in the Appendix)^2^.

These “first-appearance” TUT data are presented in Figure 1. The PG-cued TUT rate (*M* = 0.288, *SD* = 0.370) was significantly higher than was the OG-cued (*M* = 0.214, *SD* = 0.337), *t*_(251)_ = 2.82, *p* = 0.005. Just as in the previous analyses of the full data set, this numerically substantial TUT-rate difference between PG and OG cue conditions, *M* = 0.074, 95% CI [0.022, 0.125], reflected only a small effect size (*d* = 0.178), perhaps due to the inherent noise resulting from fewer data points per subject. The expected cuing effect on errors, however, was strengthened here (see Figure 2): PG-cued accuracy rate on no-go targets (*M* = 0.411, *SD* = 0.403) was significantly lower than that for OG-cued (*M* = 0.474, *SD* = 0.397) *t*_(251)_ = −2.045, *p* = 0.042. This accuracy difference, favoring OG vs. PG cues, *M* = −0.063, 95% CI [−0.125, −0.002], nonetheless represented quite a small effect (*d* = −0.129).

#### Questionnaire-based individual differences

Experiments 1–4 were not designed to assess individual differences in mind wandering and so, with sample sizes approximating 60 each, they were underpowered to detect any moderate-sized correlations (i.e., *r* ~ 0.30) among the measures (Cohen, 1992). Moreover, we selected our questionnaires primarily to mask the connection between experimental sessions, rather than to test *a priori* hypotheses about their associated constructs. With that said, these measures warrant exploratory consideration because they seem at least somewhat related conceptually to mind wandering, inattentiveness, or sustained effort and engagement: the AD/HD questionnaire asked subjects about frequency in the last 6 months for such experiences as, “*Difficulty sustaining my attention in tasks or fun activities*” and “*Easily distracted*,” the CFQ-MAL probed subjects for the 12-month frequency for items such as, “*Do you daydream when you ought to be listening to something?*” and “*Do you ‘lose your place’ in the course of carrying out some fairly routine activity?*,” and the AOS asked subjects to indicate their likely propensity among two responses to particular everyday scenarios, such as, “*When I have lost something that is very valuable to me and I can't find it anywhere: (a) I have a hard time concentrating on something else; (b) I put it out of my mind after a little while*;” and “*When I read an article in the newspaper that interests me: (a) I usually remain so interested in the article that I read the entire article; (b) I still often skip to another before I've finished the first one*.”

Higher scores on the AD/HD and CFQ-MAL measures reflected greater cognitive and behavioral difficulties, whereas higher scores on the AO scale reflected a greater action orientation (vs. state orientation). Thus, one might expect TUT rates to correlate positively with AD/HD and CFQ-MAL scores and negatively with AO scores. For completeness, Table 1 presents correlation matrices among the variables of interest for each experiment, as well as for the combined data across experiments. We comment here, however, upon only the patterns that emerged from the combined-experiment data, which reflect reasonable statistical power. First, within the SART (and consistent with prior work; e.g., McVay and Kane, 2009, 2012a), subjects with higher TUT rates failed more often to withhold responses to no-go trials and showed lower overall SART accuracy, with small-to-moderate effect sizes. Second, only the AD/HD and CFQ-MAL instruments, which correlated substantially with each other, predicted SART TUT rate, again reflecting small-to-moderate effects (higher scorers on the questionnaires demonstrated higher TUT rates; see also McVay and Kane, 2009, for similar CFQ-MAL correlations with TUTs). Third, only the CFQ-MAL, and not the AD/HD questionnaire, modestly (and negatively) predicted SART performance (these CFQ-MAL findings are also consistent with those from McVay and Kane, 2009).

**Table 1:** **Correlations among questionnaire and SART (mind wandering and performance) measures in Experiments 1–4, individually and combined**.

	**TUT**	**TACC**	***d*'**	**ADHD**	**AOS**	**CFQ**
**EXPERIMENT 1 (*N* = 63)**						
TUT	0.41 (0.219)					
TACC	−0.051	0.47 (0.190)				
*d*'	−0.108	0.861	2.57 (1.54)			
ADHD	0.174	0.182	0.025	18.56 (8.28)		
AOS	−0.071	0.017	0.097	−0.028	17.98 (2.93)	
CFQ	0.375	−0.114	−0.195	0.476	−0.161	114.46 (20.92)
**EXPERIMENT 2 (*N* = 64)**						
TUT	0.43 (0.237)					
TACC	−0.303	0.30 (0.15)				
*d*'	−0.308	0.753	1.79 (1.54)			
ADHD	0.391	0.039	−0.097	16.97 (8.87)		
AOS	−0.251	0.016	−0.002	−0.194	17.70 (3.02)	
CFQ	0.348	−0.019	−0.205	0.659	−0.275	113.23 (22.64)
**EXPERIMENT 3 (*N* = 57)**						
TUT	0.41 (0.218)					
TACC	−0.486	0.36 (0.19)				
*d*'	−0.514	0.691	2.80 (2.46)			
ADHD	0.354	−0.372	−0.265	17.00 (10.01)		
AOS	−0.265	0.298	0.178	−0.511	18.25 (2.74)	
CFQ	0.316	−0.447	−0.265	0.697	−0.396	111.40 (25.26)
**EXPERIMENT 4 (*N* = 67)**						
TUT	0.42 (0.212)					
TACC	−0.06	0.49 (0.22)				
*d*'	−0.144	0.895	4.14 (2.59)			
ADHD	0.136	0.136	0.174	17.03 (9.77)		
AOS	0.084	−0.166	−0.204	0.094	18.01 (5.31)	
CFQ	0.047	−0.148	−0.165	0.028	0.613	103.04 (18.83)
**COMBINED E1–E4**						
TUT	0.42 (0.219)					
TACC	−0.196	0.40 (0.206)				
*d*'	−0.246	0.799	2.84 (2.25)			
ADHD	0.244	0.015	−0.028	17.39 (9.21)		
AOS	−0.088	−0.010	−0.037	−0.095	17.98 (3.69)	
CFQ	0.256	−0.210	−0.255	0.466	0.035	110.39 (22.23)

*Means and standard deviations appear on the diagonal. TUT, task-unrelated thought; TACC, accuracy rate on target (no-go) trials; d', signal-detection sensitivity statistic; ADHD, sum of responses to AD/HD Rating Scale; AOS, sum of responses to Action Orientation Scale; CFQ, sum of responses on Cognitive Failures Questionnaire—Memory and Attention Lapses*.

## General discussion

Our findings are exciting but preliminary. In four experiments, we assessed subjects' personal goals and concerns during one laboratory session (embedded within other assessments) and then, in a separate session, cued those concerns with representative word triplets during an ongoing perceptual-discrimination go/no-go task. Based on current concerns theory of thought flow (e.g., Klinger, 1971, 1999, 2009), we expected that the cues to concerns (or “PG” cues) would affect the contents of subjects' thoughts. We assessed such thoughts via periodic thought probes, which asked subjects to report whether their immediately preceding thoughts were on-task or off-task. In fact, we found that PG cues elicited a subsequent, local boost in mind wandering compared to control (“OG”) cues, that is, word triplets reflecting goals not held by the subject. Moreover, because PG cues were relatively subtle and were embedded within a stream of stimuli that subjects did not have to process semantically, it is unlikely that their apparent effect on TUTs was driven by demand characteristics.

On one hand, then, our efforts to develop a class of imperative stimuli to initiate mind wandering experiences on-demand were successful, perhaps remarkably so. On the other hand, the local cuing effect we observed was small in magnitude—representing only a 3–4% increase in TUT rate following PG cues vs. OG cues—and it was not generally powerful enough to bring about corresponding disruptions to task performance (although our *post-hoc* analyses suggested that this accuracy effect may arise at subjects' *initial* encounters with PG cues). On balance, then, we suggest that implicit current-concern cuing during an ongoing task shows promise as a means by which investigators may harness the power of experimental manipulation of TUTs in order to make more substantial theoretical progress in understanding the control of thought.

Challenges remain, however. Most obvious is that people's unfulfilled goals are all different, and so it is unlikely that any “mass produced” generic stimulus set will successfully prime a large group of experimental subjects' concerns, and thereby their thoughts. Experimenters who are interested in priming mind wandering experiences are thus likely to be required, as we were, to individually create appropriate cuing stimuli from each subject's goal assessments. This is not only a time-consuming process, but it also invites the risk that some experimenters are simply more skilled or successful in generating evocative PG stimuli than are others (e.g., we do not know whether the present first author may happen to be unusually good or unusually bad at this). Clearly, such potential experimenter-degrees-of-freedom may make it challenging to evaluate future replication attempts (Simmons et al., 2011). They also make plain, however, that strong replication support from independent laboratories is necessary before this methodology can be useful in answering important theoretical questions about mind wandering. We are comforted by related findings from the goal-cuing and spontaneous-autobiographical-memory literatures (e.g., Nikula et al., 1993; Schlagman and Kvavilashvili, 2008), but some of those findings may have been influenced by demand characteristics; we also cannot be sure, of course, that those literatures are not missing some unpublished “failures” consigned to the file drawer.

Another significant hurdle to following-up on our results is that the small magnitude of the PG cuing effect will require large samples to be reliably detected. Moreover, unless these effect sizes can be meaningfully increased, it will be difficult to test for statistical interactions of PG cuing with other experimental variables of theoretical interest. We can imagine a number of possible methods that might increase the PG cuing effect, such as priming a greater number of current concerns per subject, or using longer and less ambiguous word strings to cue those concerns, or even beginning the second session with a “booster” task of having subjects write again about their to-be-primed concerns (e.g., about some impediments to their completion or some potential consequences of failure). Investigators will have to be mindful, however, that any such booster method may also increase subjects' awareness of the connection between their expressed concerns and the primes, thereby increasing the potential influence of demand characteristics on responding, particularly to the subjective reports provided at thought probes.

Despite the problems that remain, we hope the field will eventually find PG cuing to be a useful methodology in testing theories regarding the instigation, duration, termination, and regulation of off-task thought. Perhaps in the future it will be as common for researchers of mind wandering to employ imperative stimuli for their phenomena of interest as it is for researchers of other, more externally focused, phenomena of attention and consciousness (Smallwood, 2013).

### Conflict of interest statement

The authors declare that the research was conducted in the absence of any commercial or financial relationships that could be construed as a potential conflict of interest.

## Footnotes

^1^A fixed-effects model was most appropriate given the nearly identical methods and subject samples across experiments. For completeness, however, we note that a random-effects model yielded a similar point estimate for the mean PG-OG difference in TUT rate, of 0.044 [95% CI = 0.013, 0.074].

^2^In Experiment 2 only, the first appearance of one PG cue and one OG cue were followed by non-target probes. Although we did not analyze these non-target data for our main analyses, we retained them here in order to have four data-points per subject for the TUT analyses. Of course, for the target accuracy from Experiment 2, we could use only the one PG-cued trial and the one OG-cued trial, limiting each subject's contribution here to one PG trial and one OG trial.

## Appendix

Stimulus order for personal concern and control cue words, by experiment.

*Note: Each set of 45 trials repeated in succession three times in each 135-trial block. Two personal goal word triplets and two other goal triplets alternated each block (e.g., personal goal “eat more fish” would appear in Blocks 1, 3, 5, 7 and “locate new apartment” in Blocks 2, 4, 6, 8). Bolded trials reflect the first appearance of each triplet in the experiment; these were analyzed in the post-hoc analyses of “first appearance” stimuli. NT, non-target (no-go) trial; T, target (go) trial; PG, personal-goal word; OG, another subject*'*s personal-goal word; RNG, repeating non-goal word*.

**Experiment 1:**

**Block 1 set**	**Block 2 set**	**Block 3set**	**Block 4 set**	**Block 5 set**	**Block 6 set**
NT	NT	RNG1	NT	RNG1	NT
NT	NT	RNG1	NT	RNG1	NT
NT	T	RNG1	NT	RNG1	T
**RNG1**	NT	NT	NT	NT	OG2
**RNG1**	NT	NT	NT	NT	OG2
**RNG1**	NT	NT	T	NT	OG2
**NT**	OG2	T(probe)	OG2	NT	NT
**T(probe)**	OG2	NT	OG2	NT	NT
NT	OG2	NT	OG2	T(probe)	NT
NT	NT	NT	NT	NT	T(probe)
NT	T(probe)	T	NT	NT	NT
NT	NT	NT	NT	NT	NT
NT	**RNG2**	NT	NT	NT	NT
T	**RNG2**	NT	NT	T	NT
NT	**RNG2**	NT	T(probe)	NT	NT
NT	**NT**	NT	NT	NT	NT
NT	**NT**	NT	NT	NT	RNG2
NT	**NT**	NT	NT	NT	RNG2
OG1	**NT**	NT	NT	OG1	RNG2
OG1	**NT**	NT	T	OG1	NT
OG1	**T(probe)**	NT	NT	OG1	T(probe)
NT	NT	T	NT	NT	NT
NT	NT	NT	NT	NT	NT
NT	NT	NT	NT	NT	NT
NT	T	NT	NT	T(probe)	NT
NT	NT	NT	NT	NT	NT
T(probe)	NT	OG1	RNG2	NT	NT
NT	NT	OG1	RNG2	NT	NT
NT	NT	OG1	RNG2	NT	NT
NT	NT	NT	NT	NT	T
T	NT	T(probe)	NT	PG1	NT
NT	NT	NT	NT	PG1	NT
NT	NT	NT	T(probe)	PG1	NT
NT	NT	NT	NT	NT	NT
NT	NT	NT	NT	T(probe)	PG2
**PG1**	NT	NT	NT	NT	PG2
**PG1**	**PG2**	PG1	T	NT	PG2
**PG1**	**PG2**	PG1	NT	NT	NT
**NT**	**PG2**	PG1	NT	NT	NT
**NT**	**NT**	NT	PG2	NT	NT
**NT**	**NT**	NT	PG2	NT	NT
**T(probe)**	**NT**	NT	PG2	NT	NT
NT	**T(probe)**	NT	NT	T	T(probe)
NT	NT	NT	T(probe)	NT	NT
NT	NT	T(probe)	NT	NT	NT

**Experiment 2:**

**Block 1 set**	**Block 2 set**	**Block 3set**	**Block 4 set**	**Block 5 set**	**Block 6 set**	**Block 7 set**	**Block 8 set**
NT	NT	NT	NT	NT	NT	NT	NT
NT	NT	T	NT	NT	T	NT	NT
NT	NT	NT	NT	NT	NT	NT	NT
NT	NT	OG1	NT(probe)	NT(probe)	OG2	NT	NT
NT	NT	OG1	NT	NT	OG2	NT	NT
NT	**OG2**	OG1	NT	NT	OG2	OG1	NT
NT	**OG2**	NT	T	T	NT	OG1	NT
NT	**OG2**	NT(probe)	NT	NT	NT(probe)	OG1	NT
NT	**NT**	NT	NT	NT	NT	NT	NT
T(probe)	**NT(probe)**	NT	NT	NT	NT	NT(probe)	T(probe)
NT	NT	NT	NT	NT	NT	NT	NT
**PG1**	NT	NT	NT	NT	NT	NT	PG2
**PG1**	T	NT	NT	NT	NT	T	PG2
**PG1**	NT	NT	NT	NT	NT	NT	PG2
**NT**	NT	NT	NT	NT	NT	NT	NT
**NT(probe)**	NT	T	NT	NT	T	NT	NT(probe)
NT	NT	NT	NT	NT	NT	NT	NT
NT	NT	NT	T	T	NT	NT	NT
NT	T	NT	NT	NT	NT	T	NT
NT	NT	NT	PG2	PG1	NT	NT	NT
NT	NT	NT	PG2	PG1	NT	NT	NT
NT	NT	T	PG2	PG1	T	NT	NT
NT	NT	NT	NT	NT	NT	NT	NT
NT	NT	NT	NT(probe)	NT(probe)	NT	NT	NT
T	NT	PG1	NT	NT	PG2	NT	T
NT	NT(probe)	PG1	NT	NT	PG2	NT(probe)	NT
NT	NT	PG1	NT	NT	PG2	NT	NT
NT	NT	NT	NT	NT	NT	NT	NT
NT	NT	T(probe)	NT	NT	T(probe)	NT	NT
T	**PG2**	NT	OG2	OG1	NT	PG1	T
NT	**PG2**	NT	OG2	OG1	NT	PG1	NT
NT	**PG2**	NT	OG2	OG1	NT	PG1	NT
NT	**NT**	NT	NT	NT	NT	NT	NT
NT	**T(probe)**	NT	T(probe)	T(probe)	NT	T(probe)	NT
**OG1**	NT	NT	NT	NT	NT	NT	OG2
**OG1**	NT	NT	NT	NT	NT	NT	OG2
**OG1**	NT	T	T	T	T	NT	OG2
**NT**	NT	NT	NT	NT	NT	NT	NT
**T(probe)**	NT	NT	NT	NT	NT	NT	T(probe)
NT	NT	NT	NT	NT	NT	NT	NT
NT	T	T(probe)	NT	NT	T(probe)	T	NT
NT	NT	NT	T	T	NT	NT	NT
NT	NT	NT	NT	NT	NT	NT	NT
T	NT	NT	NT	NT	NT	NT	T
NT	NT	NT	NT	NT	NT	NT	NT

**Experiment 3:**

**Block 1 set**	**Block 2 set**	**Block 3set**	**Block 4 set**	**Block 5 set**	**Block 6 set**	**Block 7 set**	**Block 8 set**
NT	NT	NT	NT	NT	NT	NT	NT
NT	NT	NT	NT	NT	NT	NT	NT
NT	NT	NT	NT	NT	NT	NT	NT
NT	NT	OG1	NT	NT	OG2	NT	NT
NT	NT	OG1	NT	NT	OG2	NT	NT
NT	**OG2**	OG1	NT	NT	OG2	OG1	NT
NT	**OG2**	NT	T(probe)	T(probe)	NT	OG1	NT
NT	**OG2**	T(probe)	NT	NT	T(probe)	OG1	NT
NT	**NT**	NT	NT	NT	NT	NT	NT
NT	**T(probe)**	NT	NT	NT	NT	NT(probe)	NT
NT	NT	NT	NT	NT	NT	NT	NT
**PG1**	NT	NT	NT	NT	NT	NT	PG2
**PG1**	T	NT	NT	NT	NT	T	PG2
**PG1**	NT	NT	NT	NT	NT	NT	PG2
**NT**	NT	NT	NT	NT	NT	NT	NT
**T(probe)**	NT	T	NT	NT	T	NT	T(probe)
NT	NT	NT	NT	NT	NT	NT	NT
NT	NT	NT	NT	NT	NT	NT	NT
NT	T	NT	NT	NT	NT	T	NT
NT	NT	NT	PG2	PG1	NT	NT	NT
NT	NT	NT	PG2	PG1	NT	NT	NT
NT	NT	NT	PG2	PG1	NT	NT	NT
NT	NT	NT	NT	NT	NT	NT	NT
NT	NT	NT	T(probe)	T(probe)	NT	NT	NT
T	NT	PG1	NT	NT	PG2	NT	T
NT	NT	PG1	NT	NT	PG2	NT	NT
NT	NT	PG1	NT	NT	PG2	NT	NT
NT	NT	NT	NT	NT	NT	NT	NT
NT	NT	T(probe)	NT	NT	T(probe)	NT	NT
T(probe)	**PG2**	NT	OG2	OG1	NT	PG1	T(probe)
NT	**PG2**	NT	OG2	OG1	NT	PG1	NT
NT	**PG2**	NT	OG2	OG1	NT	PG1	NT
NT	**NT**	NT	NT	NT	NT	NT	NT
NT	**T(probe)**	NT	T(probe)	T(probe)	NT	T(probe)	NT
**OG1**	NT	NT	NT	NT	NT	NT	OG2
**OG1**	NT	NT	NT	NT	NT	NT	OG2
**OG1**	NT	T	T	T	T	NT	OG2
**NT**	NT	NT	NT	NT	NT	NT	NT
**T(probe)**	NT	NT	NT	NT	NT	NT	T(probe)
NT	NT	NT	NT	NT	NT	NT	NT
NT	T(probe)	T(probe)	NT	NT	T(probe)	T(probe)	NT
NT	NT	NT	T	T	NT	NT	NT
NT	NT	NT	NT	NT	NT	NT	NT
T	NT	NT	NT	NT	NT	NT	T
NT	NT	NT	NT	NT	NT	NT	NT

**Experiment 4:**

**Block 1 set**	**Block 2 set**	**Block 3set**	**Block 4 set**	**Block 5 set**	**Block 6 set**
NT	NT	NT	NT	NT	NT
NT	NT	NT	NT	NT	NT
NT	T	NT	NT	NT	T
NT	NT	NT	NT	NT	OG2
NT	NT	NT	NT	NT	OG2
NT	NT	NT	T	NT	OG2
NT	**OG2**	T(probe)	OG2	NT	NT
T(probe)	**OG2**	NT	OG2	NT	NT
NT	**OG2**	NT	OG2	T(probe)	NT
NT	**NT**	NT	NT	NT	T(probe)
NT	**T(probe)**	T	NT	NT	NT
NT	NT	NT	NT	NT	NT
NT	NT	NT	NT	NT	NT
T	NT	NT	NT	T	NT
NT	NT	NT	T(probe)	NT	NT
NT	NT	NT	NT	NT	NT
NT	NT	NT	NT	NT	NT
NT	NT	NT	NT	NT	NT
**OG1**	NT	NT	NT	OG1	NT
**OG1**	NT	NT	T	OG1	NT
**OG1**	T(probe)	NT	NT	OG1	T(probe)
**NT**	NT	T	NT	NT	NT
**NT**	NT	NT	NT	NT	NT
**NT**	NT	NT	NT	NT	NT
**NT**	T	NT	NT	T(probe)	NT
**NT**	NT	NT	NT	NT	NT
**T(probe)**	NT	OG1	NT	NT	NT
NT	NT	OG1	NT	NT	NT
NT	NT	OG1	NT	NT	NT
NT	NT	NT	NT	NT	T
T	NT	T(probe)	NT	PG1	NT
NT	NT	NT	NT	PG1	NT
NT	NT	NT	T(probe)	PG1	NT
NT	NT	NT	NT	NT	NT
NT	NT	NT	NT	T(probe)	PG2
**PG1**	NT	NT	NT	NT	PG2
**PG1**	**PG2**	PG1	T	NT	PG2
**PG1**	**PG2**	PG1	NT	NT	NT
**NT**	**PG2**	PG1	NT	NT	NT
**NT**	**NT**	NT	PG2	NT	NT
**NT**	**NT**	NT	PG2	NT	NT
**T(probe)**	**NT**	NT	PG2	NT	NT
NT	**T(probe)**	NT	NT	T	T(probe)
NT	NT	NT	T(probe)	NT	NT
NT	NT	T(probe)	NT	NT	NT
